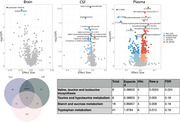# Multi‐tissue metabolomics analyses identify disrupted processes in Alzheimer’s Disease

**DOI:** 10.1002/alz.090813

**Published:** 2025-01-09

**Authors:** Ciyang Wang, Jigyasha Timsina, Muhammad Ali, Gyujin Heo, Menghan Liu, Agustin Ruiz, Pau Pastor, Yun Ju Sung, Carlos Cruchaga

**Affiliations:** ^1^ Washington University School of Medicine, Saint Louis, MO USA; ^2^ NeuroGenomics & Informatics Center, St. Louis, MO USA; ^3^ Division of Biology and Biomedical Sciences, Washington University in St. Louis, St. Louis, MO USA; ^4^ NeuroGenomics & Informatics Center, Washington University School of Medicine, St. Louis, MO USA; ^5^ Department of Psychiatry, Washington University, St. Louis, MO USA; ^6^ NeuroGenomics and Informatics Center, Washington University, St. Louis, MO USA; ^7^ Department of Psychiatry, Washington University School of Medicine, St. Louis, MO USA; ^8^ Research Center and Memory Clinic, Fundació ACE Institut Català de Neurociències Aplicades ‐ Universitat Internacional de Catalunya (UIC), Barcelona Spain; ^9^ Department of Neurology, Hospital Universitari Mutua de Terrassa, Terrassa, Barcelona Spain; ^10^ NeuroGenomics and Informatics Center, Washington University School of Medicine, St. Louis, MO USA; ^11^ Division of Biostatistics, Washington University in St. Louis, St. Louis, MO USA; ^12^ Hope Center for Neurological Disorders, Washington University School of Medicine, St. Louis, MO USA

## Abstract

**Background:**

Brain^1,2^, cerebrospinal fluid (CSF)^3,4^, and plasma^5,6^ metabolomics have been informative in identifying disrupted metabolism pathways in Alzheimer’s disease (AD). However, many AD‐focused metabolomics studies profiled a relatively small number of individuals and metabolites^3–6^, especially for CSF. In addition, past studies were limited to one or two tissues. Here we present a large‐scaled three‐tissue metabolomics study for Alzheimer’s disease.

**Method:**

The cohorts of our study include Knight‐ADRC, DIAN, ADNI, Barcelona‐1, and Fundació ACE. In this study, 362 brain samples (N.ctrl = 27, N.AD = 335), 1,974 CSF samples (N.ctrl = 855, N.AD = 1,119), and 2,369 plasma samples (N.ctrl = 1,274, N.AD = 1,095) were included. The HD4 Metabolon platform was used to measure metabolomics of plasma, CSF, and brain samples. There were 797, 456, and 1,508, metabolites quantified in brain, CSF and plasma tissues. Linear regression model including age, sex, post‐mortem interval was used to identify differentially present metabolites. FDR correction was applied to the analyzed metabolites.

**Result:**

The analyses in brain, CSF, and plasma identified 3, 65, and 553 metabolites differentially presented in AD compared to healthy controls after FDR multiple‐test correction. There were 31 metabolites shared between plasma and CSF, in which 28 having consistent direction of effects. Of the 31 CSF‐plasma shared metabolites, four metabolites (3‐hydroxyisolutyrate, alpha‐hydroxyisovalerate, ergothionenine, guaiacol sulfate), belonging to the branched‐chain amino acids (BCAAs) or xenobiotics, were nominally significant in brain. The two BCAA‐related metabolites, 3‐hydroxyisolutyrate and alpha‐hydroxyisovalerate, were replicated in another brain study^1^. Higher 3‐hydroxyisolutyrate^7^ has been associated with obesity, a risk factor for AD. We found that ergothioneine level was low in AD and a recent study showed that this metabolite improved amyloid beta clearance^8^ in a mouse model. Using metabolites differentially abundant in the plasma tissue, we found BCAA biosynthesis, taurine metabolism, starch and sucrose metabolism, and tryptophan metabolism pathways being altered in AD.

**Conclusion:**

Our study performed the first multi‐tissue metabolomics analyses on Alzheimer’s disease. We identified concordant effects across tissues for many differentially abundant metabolites. The shared metabolites throughout tissues were closely linked to the Alzheimer’s disease.